# Neuro-Metabolite Changes in a Single Season of University Ice Hockey Using Magnetic Resonance Spectroscopy

**DOI:** 10.3389/fneur.2018.00616

**Published:** 2018-08-20

**Authors:** Hemali Panchal, Nico Sollmann, Ofer Pasternak, Michael L. Alosco, Philipp Kinzel, David Kaufmann, Elisabeth Hartl, Lorie A. Forwell, Andrew M. Johnson, Elaine N. Skopelja, Martha E. Shenton, Inga K. Koerte, Paul S. Echlin, Alexander P. Lin

**Affiliations:** ^1^Department of Radiology, Brigham and Women's Hospital, Harvard Medical School, Boston, MA, United States; ^2^Center for Clinical Spectroscopy, Brigham and Women's Hospital, Harvard Medical School, Boston, MA, United States; ^3^Psychiatry Neuroimaging Laboratory, Brigham and Women's Hospital, Harvard Medical School, Boston, MA, United States; ^4^Department of Neuroradiology, Klinikum rechts der Isar, Technische Universität München, Munich, Germany; ^5^TUM-Neuroimaging Center, Klinikum rechts der Isar, Technische Universität München, Munich, Germany; ^6^Department of Neurosurgery, Klinikum rechts der Isar, Technische Universität München, Munich, Germany; ^7^Department of Psychiatry, Massachusetts General Hospital, Harvard Medical School, Boston, MA, United States; ^8^Boston University Alzheimer's Disease and CTE Center, Boston University School of Medicine, Boston, MA, United States; ^9^Department of Neurology, Boston University School of Medicine, Boston, MA, United States; ^10^Department of Child and Adolescent Psychiatry, Psychosomatic and Psychotherapy, Ludwig-Maximilians-Universität, Munich, Germany; ^11^Department of Neurology, Ludwig-Maximilians-Universität, Munich, Germany; ^12^3M Centre, The University of Western Ontario, London, ON, Canada; ^13^School of Health Studies, The University of Western Ontario, London, ON, Canada; ^14^Ruth Lilly Medical Library, Indiana University, Indianapolis, IN, United States; ^15^VA Boston Healthcare System, Brockton, MA, United States; ^16^Elliott Sports Medicine Clinic, Burlington, ON, Canada

**Keywords:** magnetic resonance spectroscopy, ice hockey, sex difference, traumatic brain injury, repetitive head injury

## Abstract

**Background:** Previous research has shown evidence for transient neuronal loss after repetitive head impacts (RHI) as demonstrated by a decrease in *N*-acetylaspartate (NAA). However, few studies have investigated other neuro-metabolites that may be altered in the presence of RHI; furthermore, the relationship of neuro-metabolite changes to neurocognitive outcome and potential sex differences remain largely unknown.

**Objective:** The aim of this study was to identify alterations in brain metabolites and their potential association with neurocognitive performance over time as well as to characterize sex-specific differences in response to RHI.

**Methods:** 33 collegiate ice hockey players (17 males and 16 females) underwent 3T magnetic resonance spectroscopy (MRS) and neurocognitive evaluation before and after the Canadian Interuniversity Sports (CIS) ice hockey season 2011–2012. The MRS voxel was placed in the corpus callosum. Pre- and postseason neurocognitive performances were assessed using the Immediate Post-Concussion Assessment and Cognitive Test (ImPACT). Absolute neuro-metabolite concentrations were then compared between pre- and postseason MRS were (level of statistical significance after correction for multiple comparisons: *p* < 0.007) and correlated to ImPACT scores for both sexes.

**Results:** A significant decrease in NAA was observed from preseason to postseason (*p* = 0.001). Furthermore, a trend toward a decrease in total choline (Cho) was observed (*p* = 0.044). Although no overall effect was observed for glutamate (Glu) over the season, a difference was observed with females showing a decrease in Glu and males showing an increase in Glu, though this was not statistically significant (*p* = 0.039). In both males and females, a negative correlation was observed between changes in Glu and changes in verbal memory (*p* = 0.008).

**Conclusion:** The results of this study demonstrate changes in absolute concentrations of neuro-metabolites following exposure to RHI. Results suggest that changes in Glu are correlated with changes in verbal memory. Future studies need to investigate further the association between brain metabolites and clinical outcome as well as sex-specific differences in the brain's response to RHI.

## Introduction

Concussions and head injuries in general are a frequent occurrence in contact sports, representing the most common injury in women's ice hockey and the second most common injury in men's ice hockey (1, 2). Further, ice hockey is a contact sport that predisposes players to concussive and subconcussive repetitive head impacts (RHI) based on the inherent nature of the sport. Though females make up nearly half of all collegiate student-athletes, females are an understudied population as few studies have included female athletes or focused on the differences in outcomes between males and females (3). Previous research nonetheless suggests that females generally have worse outcomes following concussion compared to males. More specifically, females demonstrate worse performance on neurocognitive evaluation, increased symptom severity, and have a longer recovery period compared to males (4, 5). However, the impact exposures in ice hockey in females are less frequent and of a lower magnitude compared to males (6). It is thus important to elucidate potential brain alterations in both sexes to understand how the brain responds to RHI in both sexes.

Magnetic resonance spectroscopy (MRS) has been repeatedly used to detect and to characterize changes in neuro-metabolites due to different degrees of brain injury, providing insight into the underlying mechanism of even subtle changes that can occur (7–14). MRS is a non-invasive technique that allows for the detection and quantification of neuro-metabolites *in vivo*. The primary neuro-metabolites typically assessed are *N*-acetylaspartate (NAA), a marker of neuronal and axonal integrity, creatine (Cr), often used as an internal reference for comparison to other metabolites, glutamate (Glu), a marker of excitatory neurotransmission, and myoinositol (mI), a marker for gliosis (15–17). Prior research suggests decreased levels of NAA following both acute and subacute phases of concussion (8, 14, 16). Furthermore, prior research suggests an increase in choline (Cho) and reductions in mI following brain injury (14, 16, 18, 19). More recently, changes in gamma-Aminobutyric acid (GABA) and glutathione (GSH) in response to concussion have also been reported (20, 21). In addition, and based on the literature, the corpus callosum is particularly vulnerable to the biomechanical forces suffered during RHI (22). There is therefore a need to determine the alteration of brain metabolites in regions vulnerable to RHI, such as the corpus callosum.

The aim of this study was to investigate the interactions between time and sex in male and female ice hockey players regarding changes in neuro-metabolites in the corpus callosum due to RHI during a season of collegiate ice hockey. This study evaluates the absolute concentrations of NAA, Glu, Cho, Cr, and mI at baseline and postseason. We hypothesized a decrease in NAA and an increase in total Cho (glycerophosphocholine and phosphocholine) from pre- to postseason based on previous research (7, 16, 17). Additionally, we correlated changes in neuro-metabolites due to RHI to neurocognitive performance, and hypothesized sex-specific differences in the aforementioned neuro-metabolites and correlations over time. To our knowledge, there is one study using MRS to detect sex-specific alterations in absolute neuro-metabolite concentrations due to RHI (23).

## Materials and methods

### Participants and study methods

All study participants were part of the Hockey Concussion Education Project (HCEP), which was conducted during the U Sports (formerly Canadian Interuniversity Sports (CIS)) ice hockey season of 2011–2012. The HCEP used clinical examination, neurocognitive assessment via the Immediate Post-Concussion Assessment and Cognitive Test (ImPACT), and pre- and postseason magnetic resonance imaging (MRI) as well as sequential testing and imaging at three time points after any concussion among ice hockey players.

MRS data of the HCEP participants from the 2011–2012 season have previously been evaluated for a different purpose (7). In contrast to the present study, the previous publication (1) primarily focussed on concussive RHI, (2) used ratios instead of absolute neuro-metabolite concentrations, (3) only considered NAA, Glu, and mI (in relation to Cr) during data analysis, (4) did not apply partial-volume correction, and (5) did not correlate findings of neuro-metabolite changes with neurocognitive performance (7).

Written informed consent and release of medical information was obtained from all participants prior to investigations. The study was approved by a university research ethics board. The clinical data for this study are described by Echlin et al. (24). Individuals were excluded from participation in this study based on general MRI exclusion criteria (i.e., metallic implants), structural MRI abnormalities, previous eye surgery, severe cognitive impairment, and/or a history of any psychiatric or neurological diseases. Concussion was diagnosed by the team physician, using the Zürich consensus statement (25). For this study, participants that completed pre- and postseason MRS were considered.

In total, 45 ice hockey players (25 males and 20 females) were enrolled in the 2011–2012 HCEP. Among this cohort, eight males and four females were excluded in the present study for the following reasons: missing pre- or postseason MRS (six males, two females), poor scan quality in either pre- or postseason MRS (two males and one female) based on signal-to-noise ratio (SNR) less than six, linewidths greater than 0.08 ppm, and visual inspection for substational artifacts, and the incidental finding of a large arachnoid cyst (one female). Thus, 33 participants (17 males and 16 females) were included in the analyses (Table 1).

**Table 1:** Participant-related characteristics.

		**Males**	**Females**	***p*-value**
Number of players		17	16	–
Age (in years) (mean ± SD)		22.0 ± 1.4	20.2 ± 4.5	0.135
Handedness (right/left/ambidextrous)		13/3/1	15/1/0	0.364
ImPACT score (preseason testing) (mean ± SD)	Verbal memory	81.8 ± 9.5	81.2 ± 14.2	0.188
	Visual memory	91.1 ± 6.2	86.0 ± 13.4	0.012
	Visual motor speed	42.5 ± 5.1	40.7 ± 6.8	0.665
	Reaction time	0.5 ± 0.04	0.6 ± 0.1	0.180
ImPACT score (postseason testing) (mean ± SD)	Verbal memory	82.1 ± 13.0	78.6 ± 12.2	0.426
	Visual memory	91.2 ± 7.0	92.5 ± 6.9	0.830
	Visual motor speed	47.3 ± 5.2	43.6 ± 5.4	0.597
	Reaction time	0.5 ± 0.1	0.55 ± 0.1	0.797

*This table gives an overview of participant-related characteristics, including the number of male and female participants, age, handedness, and pre- and postseason scores according to the four composite scores (verbal memory, visual memory, visual motor speed, and reaction time) derived from the results of the Immediate Post-concussion Assessment and Cognitive Test (ImPACT). Results are shown as absolute numbers or mean ± standard deviation (SD). One female participant did not undergo neurocognitive assessment by the ImPACT. The only statistically significant finding was that females had a lower preseason visual memory score compared to males (p = 0.012)*.

### Neurocognitive testing

Neurocognitive function was assessed using the ImPACT at two time points: before the season and after the hockey-playing season ends. The ImPACT is a computer-based assessment composed of a concussion symptom inventory as well as modules for assessment of neurocognitive function. Based on the results from the neurocognitive test modules, four composite scores were generated (verbal memory, visual memory, visual motor speed, and reaction time). ImPACT composite scores have been used in previous investigations among the 2011–2012 HCEP participants (26). The ImPACT results were independently evaluated by a neuropsychologist.

### Acquisition of magnetic resonance spectroscopy

Data acquisition was performed using a 3T MRI machine (Achieva 3T, Philips Medical Systems, The Netherlands B.V.) equipped with an eight-channel SENSE head coil array. Each player involved in this study received a baseline and postseason MRI evaluation. Athletes who sustained a concussion underwent additional imaging at 72 h, 2 weeks, and 2 months post-injury. This additional imaging data is not part of the current analysis.

The corpus callosum was chosen as the MRS region of interest because it forms the highest-density commissural white matter bundle in the brain. Additionally, it provides connections between hemispheres that project to the cerebrum. Furthermore, the corpus callosum is suspected to be vulnerable to damage from the biomechanical forces involved in concussion and is more predictive of outcomes compared to cortical regions (22, 27–30). The placement of the voxel within the corpus callosum also ensured an adequate distance from the ventricles, fatty tissue, and bone.

**Figure 1:**
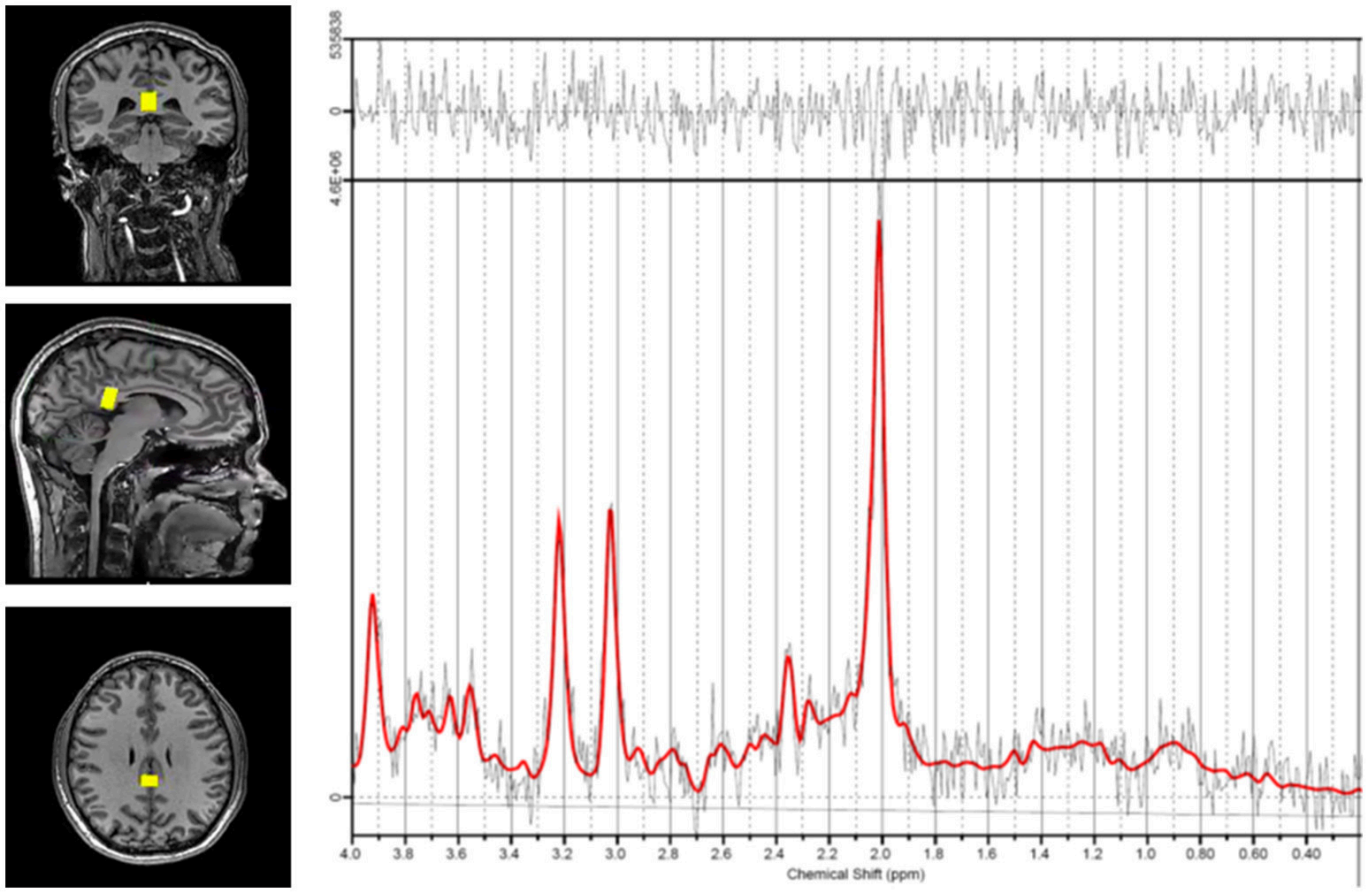
Representative spectrum and voxel location. Voxel positioning in the corpus callosum is shown on the left. The spectrum derived from measurements in this region is shown on the right. The *red* line indicates the basis set fit of the acquired data (*black* lines) and the bar at the top shows the residual difference between the fit and the spectrum.

Each patient was placed in the MRI machine and underwent a standard localizer and SENSE calibration scan. Voxels were applied for the corpus callosum (10 × 20 × 30 mm). Spectroscopic examination was carried out using a Point RESolved Spectroscopy (PRESS) pulse sequence with the following settings: echo time (TE) = 35 ms, repetition time (TR) = 2,000 ms, 128 acquisitions, and 1,024 points. Each voxel underwent automated optimization including three-dimensional shimming, transmit gain, and water suppression. A representative spectrum and corresponding voxel location is shown in Figure 1.

### Analysis of magnetic resonance spectroscopy

Phase drift correction and frequency correction were performed prior to analysis with a linear combination model. A linear combination model was also used for metabolite quantification (31). The operator-independent spectral analysis software estimates metabolite concentrations using a set of basis reference spectra acquired from individual metabolites on the MRI instrument and using the water reference spectrum for quantitation. Concentrations are derived from the areas under the corresponding peaks. Cramer-Rao lower bounds (CRLB) were calculated for each neurochemical estimation. Only those metabolites with a CRLB less than 20% were used for data analysis. The following neurochemicals were quantified: NAA, Glu, Cho, Cr, and mI. It should be noted that while Glu CRLB was low, it cannot be ensured that glutamine did not contribute to some extent to the Glu resonance though its contribution is likely to be minimal. Concentrations were partial-volume corrected by segmenting gray matter, white matter, and cerebrospinal fluid (CSF) within the voxel by mapping the voxel onto the T1-weighted image and extracting their volumes. Water concentrations were corrected by multiplying by the following factor:

[(55,556/35,880) × (1.000 × partial volume fraction of CSF) + (0.779 × partial volume of gray matter) + (0.645 × partial volume of white matter)]

This equation corrects for the assumed LCModel water concentration (WCONC; 35,880 mM) with the actual water concentration (55,556 mM), and then corrects for partial volume of each compartment of water, where CSF is assumed 100% water content, gray matter is 77.9%, and white matter is 64.5%. T1 and T2 water relaxation times at 3T (1.0 for CSF, 0.779 for gray matter, and 0.645 for white matter) are incorporated into this equation to correct for the water attenuation in LCmodel (ATTH20) as described previously (32).

### Statistical analyses

All statistical analyses were performed using SPSS (version 24; IBM SPSS Statistics for Windows, IBM Corp., Armonk, NY, USA). The MRS metabolites were examined as absolute concentrations. Repeated measures analysis of covariance (rmANCOVA) was used to examine pre- to postseason changes in the mean concentrations of each MRS metabolite. Sex was included as a between-group factor, in order to examine the presence of a sex × time interaction effect. Any history of a concussion during the season of play was included as a covariate to determine whether season changes in the MRS metabolites were independent of a concussion and associated with subconcussive RHI. This rmANCOVA was conducted for each MRS metabolite and the significance level was adjusted for the family-wise error rate using a Bonferroni adjustment for multiple comparisons, resulting in a significance level set at *p* < 0.007. A series of bivariate correlations were then conducted to examine the associations among each of the MRS metabolites and the ImPACT composite scores (i.e., verbal memory, visual memory, visual motor speed, and reaction time), which were not separately corrected for multiple comparisons. Figures on results were created using GraphPad Prism (version 6.0; GraphPad Software Inc., La Jolla, CA, USA).

## Results

### Changes of neuro-metabolite concentrations

**Figure 2:**
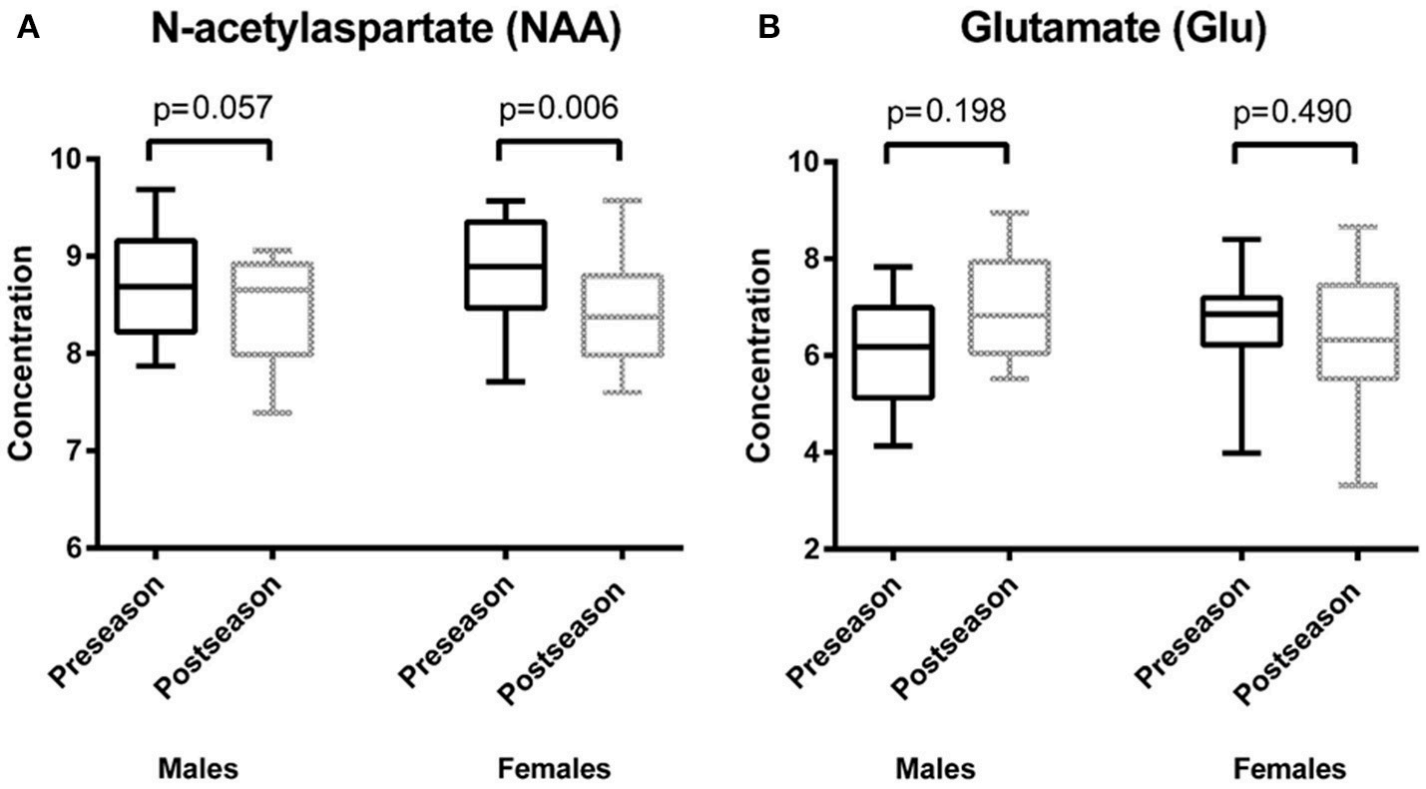
Results of repeated measures analysis of covariance **(A)** depicts box plots of average values of the concentration of *N*-acetylaspartate (NAA) at two time points: preseason and postseason. There was a statistically significant difference between pre-and postseason NAA concentrations (*p* = 0.001). In both males and females, NAA decreased when comparing the preseason to the postseason concentrations (males: *p* = 0.057, females: *p* = 0.006). **(B)** shows box plots of average values of the concentration of glutamate (Glu) at two time points: preseason and postseason. There was a trend regarding the difference between pre- and postseason Glu concentrations (*p* = 0.039). Females showed a decrease in Glu over time (*p* = 0.490), whereas males had an increase in Glu over time (*p* = 0.198).

Descriptive statistics for each metabolite at preseason and at postseason assessment are presented in Table 2. The average SNR was 8.69 for the preseason scans and 8.94 for the postseason scans. NAA was found to be significantly lower at the end of the season (postseason: 9.02 ± 0.55 mM) compared to the beginning in both males and females (preseason: 9.37 ± 0.59 mM; *p* = 0.001), regardless of whether the players had a concussion during the season (Figure 2A). Interestingly, when NAA/Cr ratio is used, there is no significant difference found (*p* = 0.228). No sex differences were observed in total change in NAA.

**Table 2:** Descriptive statistics for each neuro-metabolite at preseason and postseason.

	***NAA***		***Cr***		***Cho***		***Glu***		***mI***	
	**Mean**	***SD***	**Mean**	***SD***	**Mean**	***SD***	**Mean**	***SD***	**Mean**	***SD***
**PRESEASON**										
Male	9.29	0.61	5.05	0.76	1.53	0.17	6.57	1.15	4.49	0.97
Female	9.46	0.59	4.49	0.65	1.41	0.09	7.24	1.11	4.68	0.89
**POSTSEASON**										
Male	9.03	0.51	4.85	0.62	1.43	0.20	7.35	1.16	4.74	0.57
Female	9.01	0.61	4.53	0.46	1.39	0.17	6.76	1.41	4.75	0.87

*This table depicts mean ± standard deviation (SD) for absolute neuro-metabolite concentrations at preseason and postseason assessment considering N-acetylaspartate (NAA), creatine (Cr), choline (Cho), glutamate (Glu), and myoinositol (mI). Concentrations are measured in mM*.

A decrease in total Cho was observed, but this decrease was not statistically significant (preseason: 1.48 ± 0.16 mM, postseason: 1.41 ± 0.18 mM; *p* = 0.044). No sex differences were observed in change in total Cho. A sex effect, however, was observed for Glu, though the difference was only of trend level significance (males preseason: 6.57 ± 1.15 mM, postseason: 7.35 ± 1.16 mM; females preseason: 7.24 ± 1.11 mM, postseason: 6.76 ± 1.41 mM; *p* = 0.039), indicating that while males had an increase in Glu from pre- to postseason assessment, females had a decrease (Figure 2B). No time effect was observed from pre- to postseason MRS in regards to Glu. There was no statistically significant sex or time interactions with respect to concentrations of Cr or mI.

### Correlation of neuro-metabolite concentrations with ImPACT scores

**Figure 3:**
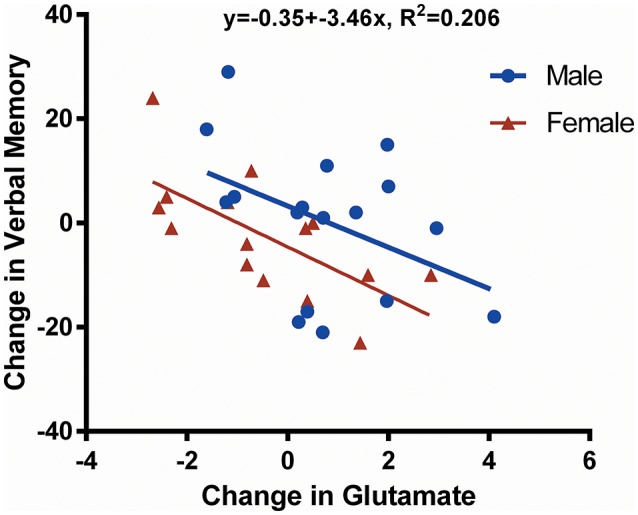
Scatter plot showing change in glutamate (Glu) against change in verbal memory. This figure illustrates the negative correlation (*R*^2^ = 0.206, *p* = 0.008) between the change in verbal memory and the change in Glu concentration over time (postseason minus preseason for males and females together). Data derived from male players are shown in *blue*, whereas data belonging to female players are shown in *red*. One female participant did not undergo neurocognitive assessment by the Immediate Post-Concussion Assessment and Cognitive Test (ImPACT); thus, only 32 data points are shown.

A negative correlation was found between change in Glu from preseason to postseason assessments and verbal memory (*p* = 0.008; Figure 3). There were no statistically significant neurocognitive differences between male and female participants except that females had a lower average visual memory score at preseason (*p* = 0.012; Table 1); however, there were no postseason differences.

## Discussion

This study found a significant decrease in NAA between pre- and postseason assessment in both male and female athletes (*p* = 0.001). A decrease in total Cho over the course of the season was observed but did not reach statistical significance when considering correction for multiple comparisons (*p* = 0.044). Females demonstrated a trend toward a decrease in Glu over the course of the play season while males demonstrated a trend toward an increase in Glu (*p* = 0.039). In both males and females, however, a negative correlation was observed between changes in Glu and changes in verbal memory (*p* = 0.008), as measured by the ImPACT.

### Changes in *N*-acetylaspartate

The significant decrease in NAA, a marker of viable neurons, from pre- to postseason was observed regardless of sex or whether the player had an in-season concussion. This result does not directly corroborate the findings of previous researchers, which have demonstrated a transient decrease in NAA after concussion, followed by a recovery in NAA (8, 33). The results of the present study suggest a persistent decrease in NAA that is still present at the end of the playing season, as opposed to a transient decrease followed by a recovery in NAA levels.

There are, however, several reasons why our findings differ from previous research. First, most previous studies investigated NAA levels in other cerebral areas, including the (dorsolateral) prefrontal cortex, posterior cingulate gyrus, or primary motor cortex (8–14). The present study investigated neuro-metabolic alterations in the corpus callosum. Therefore, it is possible that the recovery of NAA is specific to cortical areas, whereas white matter dense areas such as the corpus callosum may not be as amenable to recovery compared to cortical regions. In this context, long-coursing axons, as are present in the corpus callosum, may be more vulnerable to injury and evince less transient changes when compared to other regions (22, 30). Secondly, previous studies that demonstrated a recovery in NAA have studied concussed subjects, whereas the present study investigates both subjects with exposure to subconcussive and concussive RHI (8, 34). A previous study using participants also from the HCEP demonstrated that females who did not have an in-season concussion had a persistent decrease in NAA (i.e., at the postseason measurement), while males did not demonstrate any changes in NAA (7). One key difference between the (7) study and this study is that the NAA/Cr ratio was utilized whereas the present study used the absolute concentration of NAA (and absolute concentrations of other neuro-metabolites) (7). We chose to use absolute concentrations as opposed to the NAA/Cr ratio because it is known that Cr levels change in brain injury; therefore, we cannot distinctly assume Cr as a constant and thus should not use Cr as an internal reference standard for MRS (35, 36). As shown in the results, NAA/Cr did not show differences in this particular cohort whereas NAA concentration was significant.

The decrease in NAA levels from pre- to postseason, regardless of sex or the presence of an in-season concussion, further suggests that RHI contribute to diffuse axonal injury and neuronal loss in the corpus callosum. In this context, decreases in NAA, a marker of axonal function and integrity, are suggestive for axonal degeneration or loss (15–17, 37). Decreases in NAA are also observed in neuroinflammation and demyelination (38, 39). However, NAA alterations due to recent vigorous physical training cannot be excluded (40, 41). Thus, our results may reflect a mixed picture with the above-mentioned and other factors contributing to the observed NAA decline.

### Changes in glutamate

A trend of sex-specific difference was observed for Glu in that females had a decrease in Glu from pre- to postseason while males had an increase in Glu. This sex difference may be attributed to hormonal regulation of this excitatory neurotransmitter. The neuroprotective properties of estrogen and progesterone are well-known (42). Progesterone is thought to suppress the excitatory Glu response, while estrogen is thought to facilitate the effects of Glu transmission (43). This balance of ovarian hormones is essential for protecting the female brain from insults such as Glu excitotoxicity and oxidative stress (44, 45). Thus, the trend for a decrease in Glu in response to RHI observed in the present study may be attributed to hormonal regulation of Glu and neuroprotective properties of ovarian hormones in preventing Glu-mediated excitotoxicity.

Furthermore, previous research suggests Glu levels increase in response to injury in the male brain (11). Therefore, it is possible that the male brain does not have the hormonal neuroprotection against brain insults in the same way the female brain does. However, it is important to note that neuro-metabolite concentrations vary depending on which brain region is studied. From previous research, it is likely that Glu increases in response to brain injury, possibly potentiating aberrant neuronal signaling and propagating further signaling cascades in response to injury (11, 46). However, the regions assessed in previous research are gray matter regions; thus, the generalizability of these findings to the present study is limited. Other studies have demonstrated an increase in Glu in the splenium of the corpus callosum following mild traumatic brain injury, while a decrease in Glu in gray matter was observed (36, 47). This observation affirms the increased vulnerability of the corpus callosum to head injury, and possibly contributes to an increased likelihood of excitotoxicity in white matter regions compared to gray matter regions. However, sex differences were not investigated; thus, it is unclear if the same relationship was observed in both males and females. Furthermore, a recent study also including subjects of the HCEP demonstrated sex-dependent changes in white matter diffusivity following subconcussive head impacts, which could be due to differential hormonal regulation between males and females (48). Taken together, it is possible that hormonal regulation may play an essential role in differing levels of Glu in the corpus callosum. However, further studies are needed to elucidate the downstream consequences of increased Glu in white matter regions, and to clarify whether these downstream effects are sex-dependent.

The overall change in Glu was negatively correlated with changes in verbal memory. Previous research suggests Glu is increased in Alzheimer's disease (AD), playing a role in the pathophysiology of excitotoxicity due to an excess of *N*-methyl-D-aspartate (NMDA) receptor activation (49). The drug memantine is used for the treatment of cognitive impairment symptoms in AD and other dementias, and by acting as an NMDA receptor antagonist, it further supports the idea that Glu excitotoxicity may play a role in the cognitive symptomology of AD (50). To our knowledge, there are no studies linking an excess of Glu to a decline in verbal memory; however, it has been shown that Glu is necessary in order to perform verbal memory tasks (51, 52). Hence, an excess of Glu may be related to impaired verbal memory. This suggests an inverse relationship between excess of Glu and memory, which is in line with the findings of the present study.

### Changes in choline

A decrease, though not statistically significant, was seen in Cho, a marker of cell turnover, from pre- to postseason. Previous studies have demonstrated conflicting evidence for how Cho changes in brain injury. Many studies have reported an increase in Cho in response to brain injury, suggesting tissue breakdown and inflammation (11, 19, 53). On the other hand, another study did not find a change in Cho due to mild traumatic brain injury (54). There is modest evidence for a decrease in Cho in a cohort of retired rugby players (21). However, further studies need to be conducted in order to elucidate the complex relationship between Cho and RHI.

### Limitations

There are several limitations to this study. First, the generalizability of our findings is limited by the small sample size and the lack of a control group which would have been helpful to determine if baseline metabolite levels differ between athletes and non-athletes and whether the NAA changes are chronic or if the changes observed may be the result of data acquisition or analysis. However, this study used a prospective study design including imaging and clinical assessment in which independent non-biased specialist physician investigators were present at each game. The study investigates a broad sample of neuro-metabolites that are not routinely used in MRS studies on brain injury, and it is one of the first studies to investigate sex-specific differences in neuro-metabolites over time.

Secondly, the ImPACT, primarily designed for the detection of concussion-related symptoms, may not be sufficiently sensitive for the detection of subtle neurocognitive alterations of subjects also exposed to subconcussive RHI. At the time of data acquisition, the ImPACT assessment represented one of the few widely accessible tools to measure specific changes in neurocognitive function; however, later studies investigated more sensitive methods to assess the effects of RHI (55–57). Future studies on RHI should utilize improved, valid, and more reliable neuropsychological measures.

Thirdly, the quality of the MRS data obtained was not optimal as each scan had a comparatively low SNR. Future studies should use a larger voxel size and increase the number of averages when acquiring MRS data to ensure high data quality. Another weakness is that by using a single voxel in the corpus callosum, we were not able to study other brain regions that may be vulnerable to RHI. Future studies need to use multiple voxel locations.

Lastly, we are not able to assess whether or not the concentrations of any of the neuro-metabolites changed following the end of the season since we do not have follow-up measurements after the postseason MRS assessments. Future longitudinal studies should include follow-up examinations at different time points after the end of a period of exposure to RHI to assess if neuro-metabolite levels remain persistently altered, or if it simply takes longer to recover after RHI. Prolonged recovery after NAA decrease due to RHI has been suggested previously (58). Furthermore, prospective studies should assess hormone levels and medications taken (particularly hormonal contraceptives) during the season to assess a possible relationship between hormonal changes and neuro-metabolite changes.

## Conclusion

This study investigated differences in the change in absolute concentrations of a broad spectrum of neuro-metabolites in athletes exposed to concussive and subconcussive RHI while playing collegiate ice hockey, and, importantly, it also evaluated sex differences in these changes. Results of this study demonstrate a significant decrease in NAA, a marker of viable neurons, following exposure to RHI in both female and male athletes. Moreover, results suggest possible sex-specific differences in changes in Glu, though not statistically significant after correction for multiple comparisons. In both sexes, a negative correlation was observed between changes in Glu and changes in verbal memory. Future studies need to investigate further the association between neuro-metabolite concentration changes and clinical outcomes as well as the association of individual hormone levels and the differences in the brain's response to RHI between male and female athletes.

## Availability of data and material

All data used for analysis are presented in the manuscript. The discussion and conclusions only rely on the data presented.

## Ethics statement

This study was carried out in accordance with the recommendations of the ethics committees at each CIS university with written informed consent from all subjects. All subjects gave written informed consent in accordance with the Declaration of Helsinki.

## Author contributions

PE is responsible for concept and design. HP, NS, OP, MA, PK DK, EH, LF, AJ, ES, MS, IK, PE, and AL were responsible for data acquisition and/or analyses, handled the acquired data, conducted statistical analysis, and performed literature research. HP, NS, IK, and AL drafted the manuscript. All authors reviewed and approved the final version of the manuscript.

### Conflict of interest statement

The authors declare that the research was conducted in the absence of any commercial or financial relationships that could be construed as a potential conflict of interest.

## Abbreviations

AD: Alzheimer's disease
Cho: choline
CIS: Canadian Interuniversity Sports
Cr: creatine
CRLB: Cramer-Rao lower bounds
CSF: cerebrospinal fluid
GABA: gamma-aminobutyric acid
Glu: glutamate
GSH: glutathione
HCEP: Hockey Concussion Education Project
ImPACT: Immediate Post-Concussion Assessment and Cognitive Test
mI: myoinositol
MRI: magnetic resonance imaging
MRS: magnetic resonance spectroscopy
NAA: *N*-acetylaspartate
NMDA: *N*-methyl-D-aspartate
PRESS: Point RESolved Spectroscopy
RHI: repetitive head impacts
rmANCOVA: repeated measures analysis of covariance
SD: standard deviation
SNR: signal-to-noise ratio
TE: echo time
TR: repetition time.
